# Supplementary material for: Movement-specific signaling is differentially distributed across motor cortex layer 5 projection neuron classes
Source: Cell Rep. 2022 May 10;39(6):110801. doi: 10.1016/j.celrep.2022.110801 (PMC9620742; doi:10.1016/j.celrep.2022.110801)
Supplement: Document S1. Figures S1–S6 and Tables S1 and S2 [file mmc1.pdf]

Cell Reports, Volume 39

Supplemental information

Movement-specific signaling is differentially distributed across motor cortex layer 5 projection neuron classes

Stephen P. Currie, Julian J. Ammer, Brian Premchand, Joshua Dacre, Yufei Wu, Constantinos Eleftheriou, Matt Colligan, Thomas Clarke, Leah Mitchell, A. Aldo Faisal, Matthias H. Hennig, and Ian Duguid

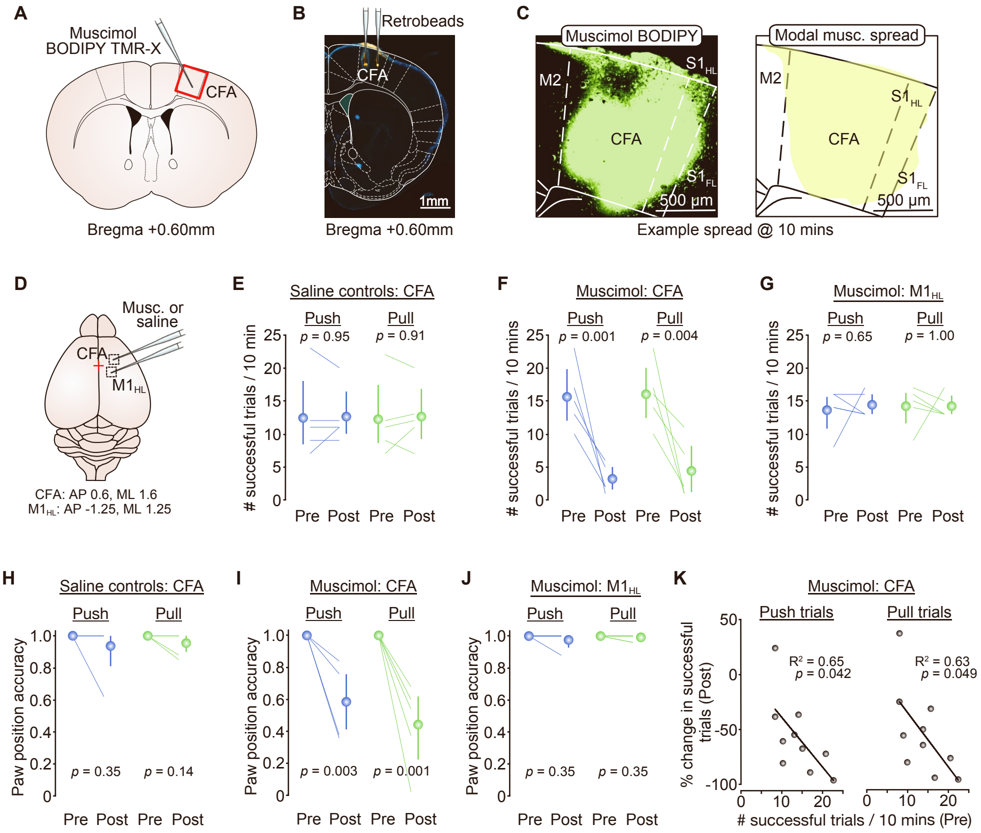

Figure S1 - Muscimol inactivation of CFA affects forelimb posture and task success. Related to Figure 1.

(A) Injection of muscimol BODIPY TMR-X into caudal forelimb area (CFA).
(B) Muscimol injection sites visualized using fluorescent retrobeads.
(C) *Left*, example image of fluorescent muscimol spread in CFA at 10 mins post injection. *Right*, modal spread of fluorescent muscimol in CFA (i.e., area in which fluorescence is present across all mice) (N = 3 mice). M2, secondary motor cortex; $S1_{HL}$, hindlimb primary somatosensory cortex; $S1_{FL}$, forelimb primary somatosensory cortex.
(D) Focal muscimol inactivation of CFA or hindlimb motor cortex ($M1_{HL}$), 0.6 mm anterior, 1.6 mm lateral of bregma and 1.25 mm posterior, 1.25 mm lateral of bregma, respectively. Red cross denotes bregma.
(E-G) Number of successful push (blue) and pull (green) trials in a 10 min period before (Pre) and after (Post) injection of (E) saline into CFA, (F) muscimol into CFA or (G) muscimol into $M1_{HL}$ (N = 5 mice), paired t-test. Colored lines, individual mice. Symbols, population means ± 95% CI.
(H-J) Paw position accuracy at the point of cue presentation before (Pre) and 10 mins after (Post) (H) saline into CFA, (I) muscimol into CFA or (J) muscimol into $M1_{HL}$ (N = 5 mice), paired t-test. Colored lines, individual mice. Symbols, population means ± 95% CI.
(K) Correlation between the number of successful push (left) and pull (right) trials before (Pre) and the % change in successful trials after (Post) muscimol injection (N = 10 mice). Symbols, individual animals, Black line, linear fit to the data (Pearson's *r*).

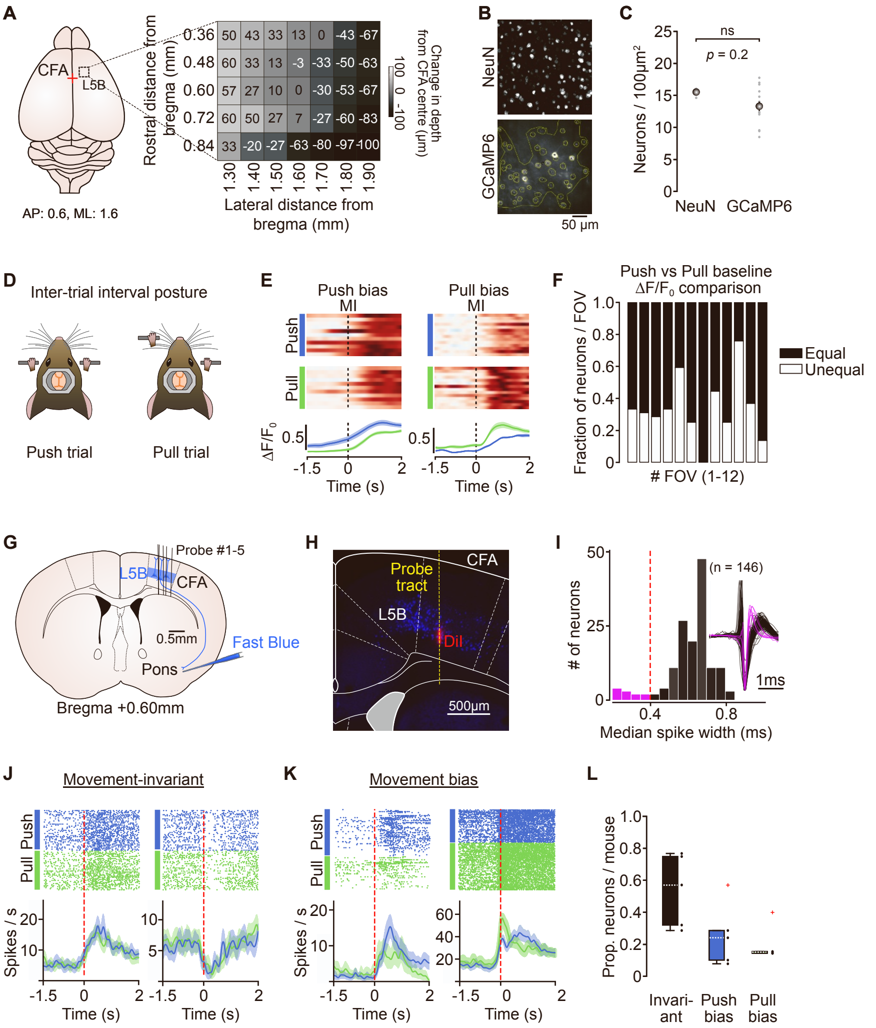

Figure S2 - Cell density and movement-type classification in layer 5B of CFA. Related to Figure 2.

(A) *Left*, schematic showing mapped region of caudal forelimb area (CFA) centred on 0.6 mm anterior, 1.6 mm medial of bregma. Red cross denotes bregma; *Right*, heatmap indicating changes in layer 5B depth across a range of cortical coordinates. Values represent the mean depth in µm of the upper boundary of Layer 5B (N = 3 mice).
(B) *Top*, representative image of NeuN stained layer 5B neurons; *Bottom*, representative two-photon FOV of L5B neurons expressing GCaMP6s. Small circles depict regions-of-interest (yellow) drawn around individual neurons within a larger bounded area excluding blood vessels.
(C) Average number of NeuN versus GCaMP6s expressing neurons / 100µm² in layer 5B of CFA. Gray dots represent individual slices, bars depict s.e.m. (NeuN, n = 3 slices, N = 1 mouse; GCaMP6s, n = 15 slices, N = 7 mice; two-sample t-test).
(D) Schematic depicting inter-trial posture for push (left) and pull (right) trials.
(E) Activity of two neurons with differences in inter-trial baseline $\Delta F/F_0$. *Left*, example neuron with push bias. *Right*, example neuron with pull bias. *Top*, raster showing normalized $\Delta F/F_0$ across successive push (blue) or pull (green) trials; *Bottom*, mean $\Delta F/F_0$ ± 95% CI for push and pull trials. Dashed lines, movement initiation (MI).
(F) Proportion of neurons per field-of-view (FOV) with equal (black) or unequal (white) inter-trial $\Delta F/F_0$ baselines (n = 486 neurons from 12 FOVs, N = 6 mice).
(G) Silicone probe recordings of putative layer 5B (L5B) neurons in CFA. Approximate position of probes within CFA are shown. The upper and lower boundaries of layer 5B are defined based on retrograde labeling of pyramidal tract (PT) neurons after FastBlue injection into the pons (N = 5 mice).
(H) Retrograde labeling of pons-projecting PT neurons within CFA (blue) with silicon probe tract visualized using DiI (red). Dashed line, overlay of probe tract.
(I) Histogram of spike durations, highlighting putative interneurons (purple) and pyramidal neurons (black). Inset, trough-aligned mean spike waveforms, normalized based on peak-to-trough height (n = 148 units, N = 5 mice).
(J) Activity of two example movement-invariant layer 5 CFA neurons. *Top*, Spike rasters during push (blue) and pull (green) trials. Dots represent individual spikes. *Bottom*, peri-stimulus time histograms depicting mean ± s.e.m. firing rate during push (blue) and pull (green) trials. Red dashed lines, movement initiation (MI).
(K) Activity of two example layer 5 CFA neurons with push (left) and pull (right) bias. *Top*, Spike rasters during push (blue) and pull (green) trials. Dots represent individual spikes. *Bottom*, peri-stimulus time histograms depicting mean ± s.e.m. firing rate during push (blue) and pull (green) trials. Red dashed lines, movement initiation (MI).
(L) Proportion of invariant, push- and pull-bias neurons per mouse (n = 71 neurons, N = 5 mice). Black dots represent individual mice. Red crosses, identified outliers.

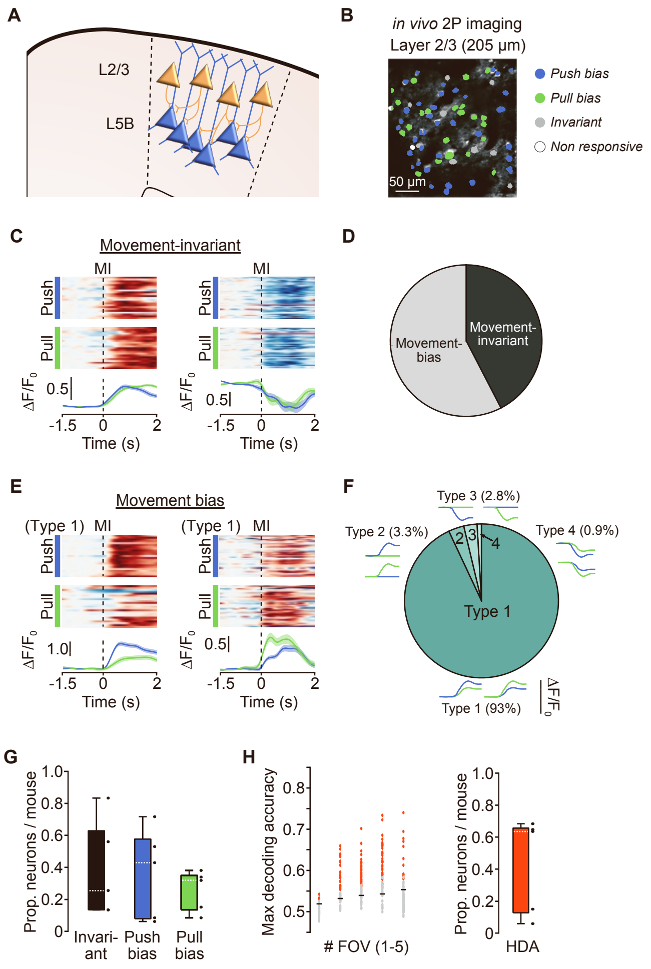

Figure S3 - Movement-specific signalling is more prevalent in Layer 2/3 neurons. Related to Figure 2 and Figure 3.

(A) Schematic showing top-down laminar inter-connectivity between layer 2/3 and layer 5B of CFA.
(B) Example FOV showing layer 2/3 CFA neurons with push (blue) or pull (green) bias. Gray, movement-invariant; white, non-responsive neurons.
(C) Activity of two example movement-invariant neurons. *Top*, raster showing normalised $\Delta F/F_0$ across successive push (blue) or pull (green) trials; *Bottom*, mean $\Delta F/F_0$ ± 95% CI for push and pull trials. Dashed lines, movement initiation (MI).
(D) Summary of movement-invariant and movement-bias neuron classification in CFA layer 2/3 neurons (n = 215 vs 160 neurons, N = 5 mice).
(E) Activity of two example Type 1 movement-bias neurons. *Top*, raster showing normalised $\Delta F/F_0$ across successive push (blue) or pull (green) trials; *Bottom*, mean $\Delta F/F_0$ ± 95% CI for push and pull trials. Dashed lines, movement initiation (MI).
(F) Summary of movement-bias classification in CFA layer 2/3 neurons (n = 215 neurons, N = 5 mice). *Insets*, model examples of $\Delta F/F_0$ changes classified as Type 1 - 4.
(G) Proportion of invariant, push- and pull-bias neurons per mouse (n = 375 neurons from 5 FOVs, N = 5 mice). Black dots represent individual mice.
(H) *Left*, maximum decoding accuracy during peri-movement epochs generated using a naïve Bayes classifier. Circles represent individual neurons; black horizontal lines indicate significance threshold. *Right*, box-and-whisker plot showing the proportion of high decoding accuracy (HDA) neurons across mice (n = 375 neurons from 5 FOVs, N = 5 mice).

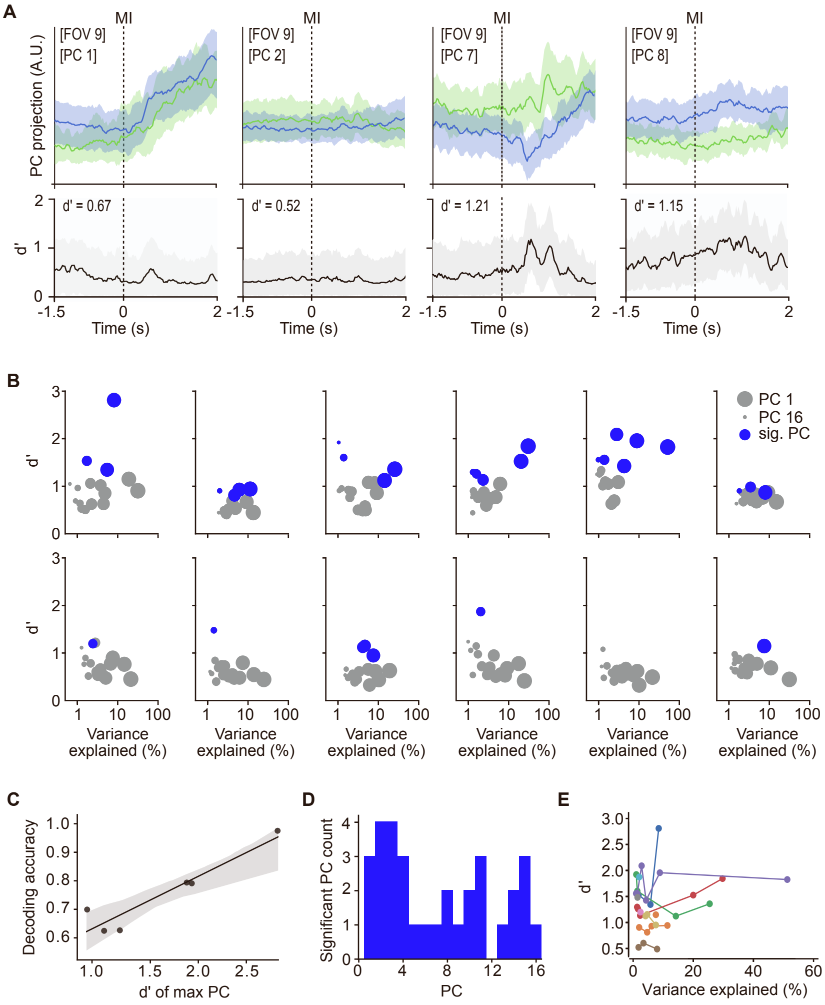

Figure S4 - Movement type is represented in both leading and higher principal components. Related to Figure 3.

(A) *Top*, example trajectories of 4 principal components (PC) from a representative field-of-view (FOV) during push (blue) and pull (green trials). *Bottom*, discrimination index (d') calculated from the corresponding PCs. Inset, maximum d' of each PC. Thick lines, mean ± 95% CI. Dashed lines, movement initiation (MI).
(B) Variance explained as a function of discrimination index (d') for all PCs in each FOV. *Top*, FOVs 1-6. *Bottom*, FOVs 7-12. Dot size (large to small) represents PC rank (1-16). Blue dots are significant PCs.
(C) Discrimination index (d') of the highest significant PC vs population decoding accuracy (Figure 3) for each mouse (N = 6 mice). Black line, linear regression fit ± 95% CI.
(D) Histogram of significant PCs across all 12 FOVs.
(E) Significance explained as a function of discrimination index (d') for all significant PCs. Dots represent individual PCs. Lines and colours link PCs from the same FOV.

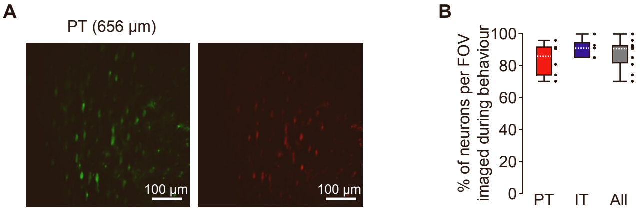

Figure S5 - Quantifying proportions of imaged neurons per field-of-view. Related to Figure 4.

(A) Example field-of-view (FOV) showing PT neurons expressing GCaMP6s (left; green) and mRuby (right; red).
(B) Percentage of mRuby positive PT and IT neurons per FOV imaged ($\Delta F/F_0$) during behaviour. Box-and-whisker plots showing median, interquartile range and range. PT (red), IT (purple) and all neurons (gray). Black dots represent individual FOVs. N = 6, 5 and 11, respectively.

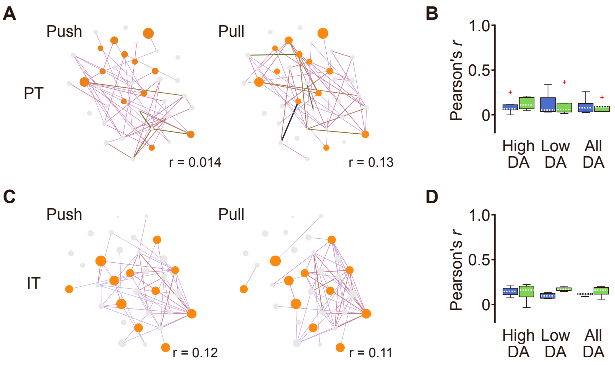

Figure S6 - Temporal organization of high decoding accuracy neurons in layer 5B during push and pull movements. Related to Figure 5.

(A) *Left & right*, functional networks constructed from the pairwise activity correlations from a representative PT field-of-view (FOV) for push (left) or pull (right) trials. Line color (light to dark) and width correspond to increasing values of Pearson's *r*. Neurons are plotted as nodes in Euclidean space with color and size relating to increasing decoding accuracy.
(B) Box-and-whisker plots showing the median, interquartile range and range of correlation coefficients across mice for high decoding accuracy (HDA), low decoding accuracy (LDA) and all neurons. Data are shown for both push (blue) and pull (green) trials. Red crosses, identified outliers.
(C) *Left & right*, functional networks constructed from the pairwise activity correlations from a representative IT FOV for push (left) or pull (right) trials. Line color (light to dark) and width correspond to increasing values of Pearson's *r*. Neurons are plotted as nodes in Euclidean space with color and size relating to increasing decoding accuracy.
(D) Box-and-whisker plots showing the median, interquartile range and range of correlation coefficients across mice for high decoding accuracy (HDA), low decoding accuracy (LDA) and all neurons. Data are shown for both push (blue) and pull (green) trials.

Table S1 - Contributions Matrix. Related to Figures 1-5 and Figures S1-S6.

	SC	JA	BP	JD	YW	CE	MC	TC	LM	AF	MH	ID
Conceived and initiated the project		Minor	Major									Major
Forelimb kinematic tracking	Major	Minor	Minor									
In vivo pharmacology	Major			Major					Minor			
Quantifying muscimol diffusion	Minor							Major				
Tracing and immunohistochemistry			Major	Minor					Minor			
Two-photon imaging	Major	Major	Major									
Imaging analysis pipelines	Minor	Major	Minor									
Extracellular recording / spike sorting				Major								
Neural decoding	Major				Major							
Dimensionality reduction											Major	
Analysed data	Major	Major	Major	Minor	Minor	Major	Major	Minor	Minor			
Produced figures	Major	Minor	Minor	Minor		Minor	Minor	Minor			Minor	Major
Managed the project												Major
Manuscript writing	Minor											Major
Discussion and interpretation	Major	Major	Major	Minor	Minor	Minor	Minor			Minor	Minor	Major

Major contribution

Minor contribution

N/A

Table S2, statistical details, related to Figures 1-5 and Figures S1-S6.

$\bar{X}$ = mean | $\tilde{X}$ = median | SD = standard deviation | IQR = interquartile range
fov = fields-of-view | H / LDA = high / low decoding accuracy | GLMM = generalised linear mixed-effects model

Figure	Description	Sample size	Result	Variance	Confidence intervals (95%)	Statistical test result
1C-E	Training time (days)	N = 24	$\tilde{X}$ = 10.5	IQR = 4		
1C	Reaction time (s; push / pull)	N = 24	$\tilde{X}$ = 0.132 / 0.135	IQR = 0.058 / 0.043		
	Movement duration (s; push / pull)	N = 24	$\tilde{X}$ = 706 / 422	IQR = 352 / 438		
1D	Successful trials / session (push / pull)	N = 24	$\tilde{X}$ = 44.5 / 45	IQR = 9.5 / 8.5		
1E	Task success (%; push / pull)	N = 24	$\tilde{X}$ = 68.0 / 74.5	IQR = 35.9 / 43.4		
1G	Successful trials / 10min (push muscimol CFA pre / post)	N = 10	$\bar{X}$ = 13.9 / 5	SD = 5.22 / 2.94	[11.1 17.1] / [3.3 6.8]	t(18) = 4.70, P = 1.79×10^{-4}, paired t-test
	Successful trials / 10min (pull muscimol CFA pre / post)	N = 10	$\bar{X}$ = 14.0 / 5.3	SD = 5.33 / 3.62	[10.9 17.2] / [3.2 7.4]	t(18) = 4.27, P = 4.64×10^{-4}, paired t-test
1H	Paw position accuracy (push muscimol CFA pre / post)	N = 10	$\bar{X}$ = 1 / 0.66	SD = 0 / 0.2	[1 1] / [0.54 0.77]	t(18) = 5.56, P = 2.79×10^{-5}, paired t-test
	Paw position accuracy (pull muscimol CFA pre / post)	N = 10	$\bar{X}$ = 0.98 / 0.50	SD = 0.06 / 0.21	[0.95 1] / [0.36 0.62]	t(18) = 6.85, P = 2.07×10^{-6}, paired t-test
S1. E	Successful trials / 10min (push saline CFA pre / post)	N = 5	$\bar{X}$ = 12.4 / 12.6	SD = 6.23 / 4.28	[8.6 18] / [10 16.4]	t(8) = -0.06, P = 0.95, paired t-test
	Successful trials / 10min (pull saline CFA pre / post)	N = 5	$\bar{X}$ = 12.2 / 12.6	SD = 5.81 / 4.72	[8.6 17.4] / [9 16.6]	t(8) = -0.12, P = 0.91, paired t-test

S1. F	Successful trials / 10min (push muscimol CFA pre / post)	N = 5	$\bar{X}$ = 15.6 / 3.2	SD = 4.88 / 2.17	[12.0 19.8] / [1.6 4.8]	t(8) = 5.19, P = 8.29x10^{-4}, paired t-test
	Successful trials / 10min (pull muscimol CFA pre / post)	N = 5	$\bar{X}$ = 16.0 / 4.4	SD = 4.74 / 4.45	[12.4 20.0] / [1.2 8.2]	t(8) = 3.99, P = 4.0x10^{-3}, paired t-test
S1. G	Successful trials / 10min (push muscimol $M1_{HL}$ pre / post)	N = 5	$\bar{X}$ = 13.6 / 14.4	SD = 3.29 / 1.95	[10.8 15.6] / [13.0 16.0]	t(8) = -0.47, P = 0.65, paired t-test
	Successful trials / 10min (pull muscimol $M1_{HL}$ pre / post)	N = 5	$\bar{X}$ = 14.2 / 14.2	SD = 3.11 / 1.79	[11.4 16.2] / [13.0 15.8]	t(8) = 0, P = 1.0, paired t-test
S1. H	Paw position accuracy (push saline CFA pre / post)	N = 5	$\bar{X}$ = 1 / 0.92	SD = 0 / 0.17	[1 1] / [0.77 1]	t(8) = 1.0, P = 0.35, paired t-test
	Paw position accuracy (pull saline CFA pre / post)	N = 5	$\bar{X}$ = 1 / 0.95	SD = 0 / 0.07	[1 1] / [0.89 1]	t(8) = 1.62, P = 0.15, paired t-test
S1. I	Paw position accuracy (push muscimol CFA pre / post)	N = 5	$\bar{X}$ = 1 / 0.59	SD = 0 / 0.22	[1 1] / [0.41 0.76]	t(8) = 4.27, P = 2.7x10^{-3}, paired t-test
	Paw position accuracy (pull muscimol CFA pre / post)	N = 5	$\bar{X}$ = 1 / 0.44	SD = 0 / 0.26	[1 1] / [0.23 0.62]	t(8) = 4.79, P = 1.4x10^{-3}, paired t-test
S1. J	Paw position accuracy (push muscimol $M1_{HL}$ pre / post)	N = 5	$\bar{X}$ = 1 / 0.98	SD = 0 / 0.05	[1 1] / [0.93 1]	1(8) = 1.0, P = 0.35, paired t-test
	Paw position accuracy (pull muscimol $M1_{HL}$ pre / post)	N = 5	$\bar{X}$ = 1 / 0.99	SD = 0 / 0.02	[1 1] / [0.98 1]	1(8) = 1.0, P = 0.35, paired t-test
S1. K	Successful trial vs % change in successful trials (push / pull)	N = 10				r^2 = -0.65 P = 0.042 / r^2 = -0.63, P = 0.049
2D	Prop. of responsive neurons (non / movement / reward)	N = 6<br>fov = 12<br>cell = 653	$\bar{X}$ = 20.9 / 73.5 / 5.7	SD = 15.1 / 16.0 / 3.7	[12.8 29.1] / [64.7 81.8] / [3.7 7.7]	

2E	Trial-trial correlation - ΔF/F0 vs motion index for push trials (motion index correlation 0.1 / 0.3 / 0.5 0.7)	N = 6<br>fov = 12<br>trials = 2931	$\tilde{X}$ = 0.36 / 0.62 / 0.75 / 0.80		[0.24 0.40] / [0.46 0.68] / [0.60 0.86] / [0.65 0.89]	
	Trial-trial correlation - ΔF/F0 vs motion index for pull trials (motion index correlation 0.1 / 0.3 / 0.5 0.7)	N = 6<br>fov = 12<br>trials = 2931	$\tilde{X}$ = 0.25 / 0.47 / 0.59 / 0.79		[0.23 0.27] / [0.37 0.87] / [0.52 0.68] / [0.65 0.89]	
2G	# of movement-responsive neurons (%; bias / invariant)	N = 6<br>fov = 12<br>Cell = 468	181 (38.7) / 287 (61.3)			
2J	# of movement bias neurons (%; type 1 / 2 / 3 / 4)	N = 6<br>fov = 12<br>Cell = 181	136 (75.1) / 25 (13.8) / 15 (8.3) / 5 (2.8)			
2L	Average ΔF/F0 trial-trial correlation for push trials (non / push / push)	N = 6<br>fov = 12<br>cell = 468	$\tilde{X}$ = 0.37 / 0.49 / 0.45	IQR = 0.37 / 0.47 / 0.39	[0.12 0.56] / [0.21 0.72] / [0.04 0.70]	
	Average ΔF/F0 trial-trial correlation for pull trials (non / push / push)	N = 6<br>fov = 12<br>cell = 468	$\tilde{X}$ = 0.31 / 0.30 / 0.53	IQR = 0.25 / 0.32 / 0.33	[0.10 0.65] / [0.08 0.75] / [0.06 0.61]	
2M	Prop. of movement-responsive neurons (non / push / pull)	N = 6<br>fov = 12<br>cell = 468	$\tilde{X}$ = 59.8 / 14.3 / 11.8	IQR = 31.4 / 15.9 / 19.5		
S2. C	Neurons / 100 μm^2 (NeuN / GCaMP6s)	N = 1 / 7<br>fov = 3 / 15	$\bar{X}$ = 15.6 / 13.3	SD = 0.9 / 2.7		t(16) = 1.75, *P* = 0.2, Student's t-test
S2. E-F	Prop. of biased neurons (equal / unequal baseline)	N = 6<br>fov = 12<br>cell = 181	$\tilde{X}$ = 69.5 / 30.5	IQR = 8.2 / 8.2		
S2. G-L	# units (%; pyramidal / interneurons)	N = 5<br>n = 146	137 (93.8) / 9 (6.2)			

	# units (%; non / move / reward)	N = 5<br>n = 137	61 (45.2) / 72 (52.6) / 4 (2.9)			
S2. L	Prop. of movement-responsive neurons (non / push / pull)	N = 5<br>n = 72	$\tilde{X}$ = 0.57 / 0.15 / 0.24	IQR = 0.43 / 0.01 / 0.19		
S3. D	# of movement-responsive neurons L2-3 (%; bias / invariant)	N = 5<br>fov = 5<br>cell = 375	216 (57.6) / 159 (42.4)			
S3. F	# of movement bias neurons L2-3 (%; type 1 / 2 / 3 / 4)	N = 5<br>fov = 5<br>cell = 216	200 (93.0) / 7 (3.3) / 6 (2.8) / 2 (0.9)			
S3. G	Prop. of movement-responsive neurons L2-3 (non / push / pull)	N = 5<br>fov = 5<br>cell = 375	$\tilde{X}$ = 0.25 / 0.43 / 0.32	IQR = 0.50 / 0.50 / 0.22		
S3. H	Prop. of movement-responsive neurons L2-3	N = 5<br>fov = 5<br>cell = 375	$\tilde{X}$ = 0.64	IQR = 0.53		
3A	Prop. of movement-responsive neurons (LDA / HDA)	N = 6<br>fov = 12<br>cell = 468	0.63 / 0.37			
3B	Decoding accuracy (single cell / population)	N = 6<br>fov = 12	$\tilde{X}$ = 0.61 / 0.75	IQR = 0.07 / 0.16		W = 1, Z = -2.20, P = 2.8×10^{-2}, Wilcoxon signed rank test
3F	Prop. neurons removed (high-low / random)	N = 6<br>fov = 9	$\tilde{X}$ = 0.21 / 0.64	IQR = 0.5 / 0.57		W = 1, Z = -2.20, P = 2.8×10^{-2}, Wilcoxon signed rank test
S4. C	Population decoding vs max d’	N = 6<br>fov = 10				r^2 =0.88, F(1,5) = 29.95, P = 5.4×10^{-3}

4E	# of movement-responsive neurons PT (%; bias / invariant)	N = 5<br>fov = 6<br>cell = 171	46 (26.9) / 125 (73.1)			
	# of movement bias neurons PT (%; type 1 / 2 / 3 / 4)	N = 5<br>fov = 6<br>cell = 46	36 (78.2) / 9 (19.6) / 1 (2.2) / 0 (0)			
	Prop. of movement-responsive neurons PT (non / push / pull)	N = 5<br>fov = 6<br>cell = 171	$\tilde{X}$ = 0.75 / 0.10 / 0.14	IQR = 0.21 / 0.19 / 0.11		
4G	# of movement-responsive neurons IT (%; bias / invariant)	N = 4<br>fov = 5<br>cell = 110	54 (49.1) / 56 (50.9)			
	# of movement bias neurons IT (%; type 1 / 2 / 3 / 4)	N = 4<br>fov = 5<br>cell = 54	34 (62.9) / 15 (27.8) / 2 (3.7) / 3 (5.6)			
	Prop. of movement-responsive neurons IT (non / push / pull)	N = 4<br>fov = 5<br>cell = 110	$\tilde{X}$ = 0.51 / 0.21 / 0.32	IQR = 0.11 / 0.26 / 0.31		
4I	Decoding accuracy PT (single / population) (HDA only)	N = 5<br>fov = 6<br>cell = 58	$\tilde{X}$ = 0.55 / 0.68	IQR = 0.02 / 0.14		t(4) = -3.04, *P* = 0.04
	Decoding accuracy IT (single / population) (HDA only)	N = 4<br>fov = 5<br>cell = 43	$\tilde{X}$ = 0.56 / 0.73	IQR = 0.04 / 0.21		t(3) = -2.95, *P* = 0.06
	Decoding accuracy population (PT / IT) (HDA only)	N = 5 / 4<br>fov = 6 / 5<br>cell = 58 / 43	$\tilde{X}$ = 0.68 / 0.73	IQR = 0.14 / 0.21		t(7) = 0.40, *P* = 0.70
S5. B	% of all neurons per FOV imaged during behaviour (PT / IT / All)	N = 5 / 4 / 9	$\tilde{X}$ = 85.8 / 91.2 / 90.7	IQR = 17.4 / 9.3 / 10.7		

		fov = 6 / 5 / 11				
5B	Norm. prop. $\Delta F/F_0$ onsets push PT (ms; HDA / LDA). ANOVA [animal:cell type: onset time]	N = 5<br>fov = 6<br>cell = 238	$\tilde{X}$ = 210 / 113	IQR = 709 / 662		F(4) = 0, *P* = 1 / F(1) = 0, *P* = 1 / F(17) = 5.12, *P* = $8.32\text{x}10^{-9}$
	Norm. prop. $\Delta F/F_0$ onsets pull PT (ms; HDA / LDA). ANOVA [animal:cell type: onset time]	N = 5<br>fov = 6<br>cell = 238	$\tilde{X}$ = 145 / 177	IQR = 226 / 500		F(3) = 0, *P* = 1 / F(1) = 0, *P* = 1 / F(17) = 8.57, *P* = $3.35\text{x}10^{-15}$
	Norm. prop. $\Delta F/F_0$ onsets push IT (ms; HDA / LDA). ANOVA [animal:cell type: onset time]	N = 4<br>fov = 5<br>cell = 137	$\tilde{X}$ = 500 / 339	IQR = 524 / 677		F(3) = $1.1\text{x}10^{-14}$, *P* = 1 / F(1) = 0, *P* = 1 / F(17) = 7.31, *P* = $4.85\text{x}10^{-12}$
	Norm. prop. $\Delta F/F_0$ onsets pull IT (ms; HDA / LDA). ANOVA [animal:cell type: onset time]	N = 4<br>fov = 5<br>cell = 137	$\tilde{X}$ = 403 / 468	IQR = 444 / 710		F(3) = 0, *P* = 1 / F(1) = 0, *P* = 1 / F(17) = 3.81, *P* = $6.85\text{x}10^{-6}$
5D	Pairwise correlation coefficient PT (r; push; HDA vs ALL / LDA vs ALL)	N = 5<br>fov = 6<br>cell = 171	HDA $\tilde{X}$ = 0.07<br>LDA $\tilde{X}$ = 0.06<br>ALL $\tilde{X}$ = 0.08	HDA IQR = 0.05<br>LDA IQR = 0.16<br>ALL IQR = 0.10	[-0.103 0.087] / [-0.085 0.094]	*P* = 0.84 / 1.0
	Pairwise correlation coefficient IT (r; push; HDA vs ALL / LDA vs ALL)	N = 4<br>fov = 5<br>cell = 110	HDA $\tilde{X}$ = 0.15<br>LDA $\tilde{X}$ = 0.09<br>ALL $\tilde{X}$ = 0.12	HDA IQR = 0.07<br>LDA IQR = 0.05<br>ALL IQR = 0.01	[-0.093 0.081] / [-0.073 0.076]	*P* = 1.0 / 0.96
5E	Pairwise corr. Vs distance PT (HDA / LDA)	N = 5<br>fov = 6<br>cell = 171	Spearman r = -0.1 / 0.0			*P* = 0.87 / 1.0
	GLMM PT<br>r distance pairwise accuracydecoder + movementtype+animal	N = 5, n = 3024 observations				*P* = 0.33
	Pairwise corr. Vs distance IT (HDA / LDA)	N = 4<br>fov = 5	Spearman r = -0.40 / 0.0			*P* = 0.6 / 1.0

		cell = 110				
	GLMM IT<br>r distance pairwise accuracydecoder + movementtype+animal	N = 5, n = 1562 observations				*P* = 0.49
5G	Pairwise distance PT (µm; HDA vs ALL / LDA vs ALL)	N = 5<br>fov = 6<br>cell = 171	HDA $\tilde{X}$ = 226<br>LDA $\tilde{X}$ = 273<br>ALL $\tilde{X}$ = 264	HDA IQR = 26<br>LDA IQR = 60<br>ALL IQR = 53	[-69 102] / [-62 69]	*P* = 1.0 / 1.0
	Pairwise distance IT (µm; HDA vs ALL / LDA vs ALL)	N = 4<br>fov = 5<br>cell = 110	HDA $\tilde{X}$ = 228<br>LDA $\tilde{X}$ = 220<br>ALL $\tilde{X}$ = 229	HDA IQR = 14<br>LDA IQR = 56<br>ALL IQR = 58	[-82 67] / [-63 68]	*P* = 0.88 / 1.0
S6. B	Pairwise correlation coefficient PT (r; pull; HDA vs ALL / LDA vs ALL)	N = 5<br>fov = 6<br>cell = 171	HDA $\tilde{X}$ = 0.11<br>LDA $\tilde{X}$ = 0.06<br>ALL $\tilde{X}$ = 0.09	HDA IQR = 0.12<br>LDA IQR = 0.10<br>ALL IQR = 0.06	[-0.1 0.15] / [-0.1 0.09]	*P* = 1.17 / 0.79
S6. D	Pairwise correlation coefficient IT (r; pull; HDA vs ALL / LDA vs ALL)	N = 4<br>fov = 5<br>cell = 110	HDA $\tilde{X}$ = 0.16<br>LDA $\tilde{X}$ = 0.17<br>ALL $\tilde{X}$ = 0.16	HDA IQR = 0.12<br>LDA IQR = 0.03<br>ALL IQR = 0.08	[-0.08 0.07] / [-0.07 0.1]	*P* = 0.89 / 1.28
